# Supplementary material for: Characteristics of PSA Bounce after Radiotherapy for Prostate Cancer: A Meta-Analysis
Source: Cancers (Basel). 2020 Aug 5;12(8):2180. doi: 10.3390/cancers12082180 (PMC7465291; doi:10.3390/cancers12082180)
Supplement: Supplementary file 1 [file cancers-12-02180-s001.pdf]

Supplementary Materials:

Characteristics of PSA Bounce After Radiotherapy For Prostate Cancer: A Meta-Analysis

Table S1. The number of studies and patients according to radiotherapy modality.

Radiotherapy Modality	Number of Studies	Number of Patients
LDR-BT	26	12753
HDR-BT	5	1240
EBRT	10	6811
SBRT	14	3573
EBRT + boost	9	1750
CIRT	1	131

LDR-BT, low dose-rate brachytherapy; HDR-BT, high dose-rate brachytherapy; EBRT, external beam radiotherapy; SBRT, stereotactic body radiotherapy; CIRT, carbon ion radiotherapy. Boost irradiation included HDR-BT, HDR-BT, and SBRT.

Table S2. Definition of endpoints.

Endpoints	Definition
Primary endpoint	
PSA bounce	PSA increase >0.2 ng/mL followed by spontaneous decrease to pre-bounce nadir
Secondary endpoint	
Bounce amplitude	Highest PSA value during PSA bounce
Time to occurrence	Post-radiotherapy duration until occurrence of PSA bounce
Nadir value	Lowest PSA value post-radiotherapy
Time to nadir	Post-radiotherapy duration until nadir

PSA, prostate-specific antigen.

Table S3. Search strategy.

Database	Search Terms	Search Strategy
Medline	PSA kinetics AND radiotherapy	Sorted by Best Match
	PSA bounce AND radiation AND therapy	Sorted by Best Match
Web of Science	PSA (bounce OR spike) AND (radiotherapy OR brachytherapy)	Limit was not applied

PSA, prostate-specific antigen.

Table S4. PICO metrics.

Metrics	Contents
Population	Prostate cancer
Intervention	Radiotherapy (any modality)
Comparison	No comparison is needed
Outcome	
Mandatory	PSA bounce (cutoff, 0.2 ng/mL)
Optional	Bounce amplitude, tune to occurrence, nadir value, time to nadir

PICO, Population-Intervention-Comparison-Outcome; PSA, prostate-specific antigen.

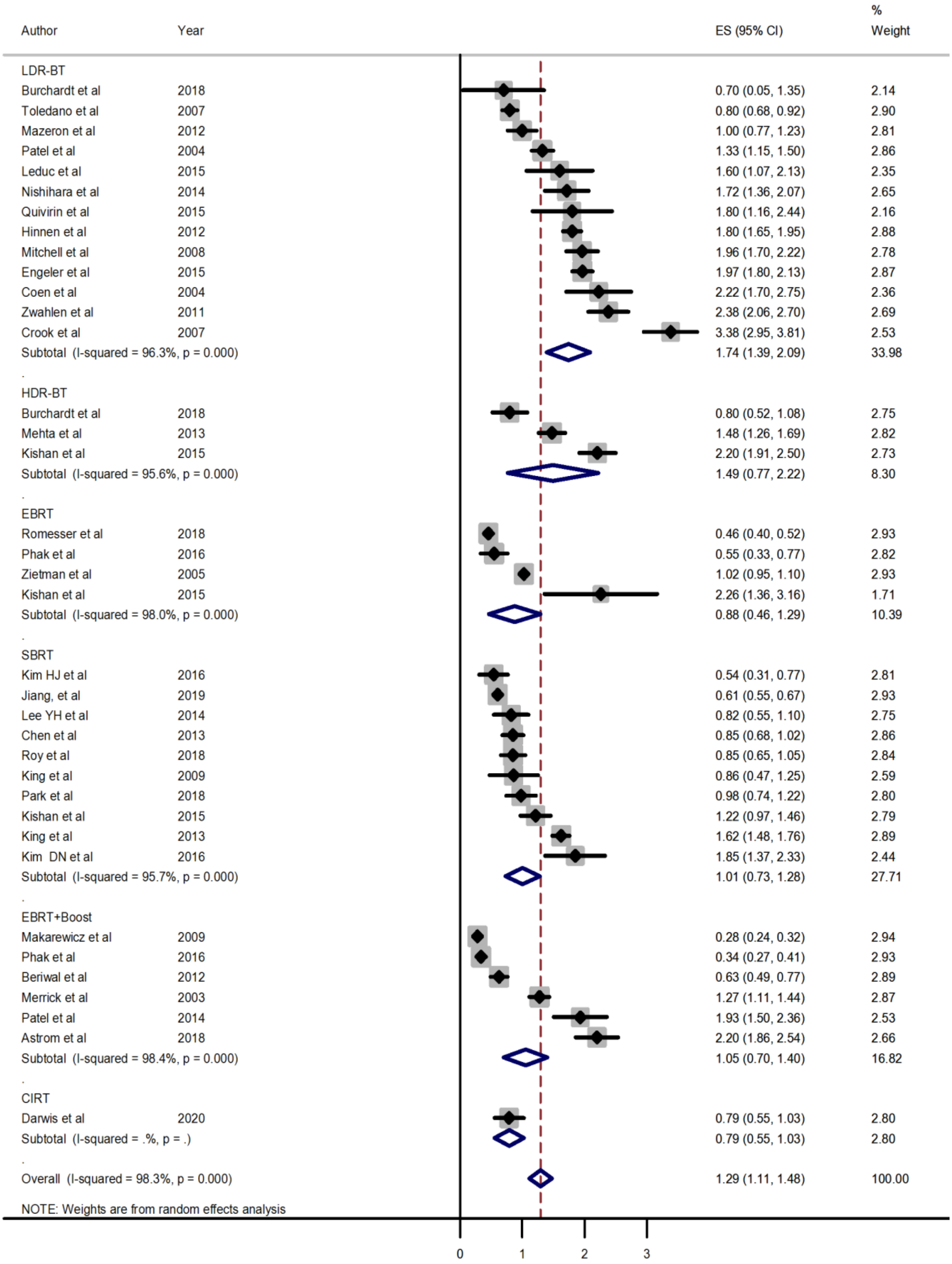

Figure S1. Meta-analysis of the amplitude of prostate-specific antigen (PSA) bounce after radiotherapy. ES, effect size; CI, confidence interval; LDR-BT, low dose-rate brachytherapy; HDR-BT, high dose-rate brachytherapy; EBRT, external beam radiotherapy; SBRT, stereotactic body radiotherapy; CIRT, carbon ion radiotherapy.

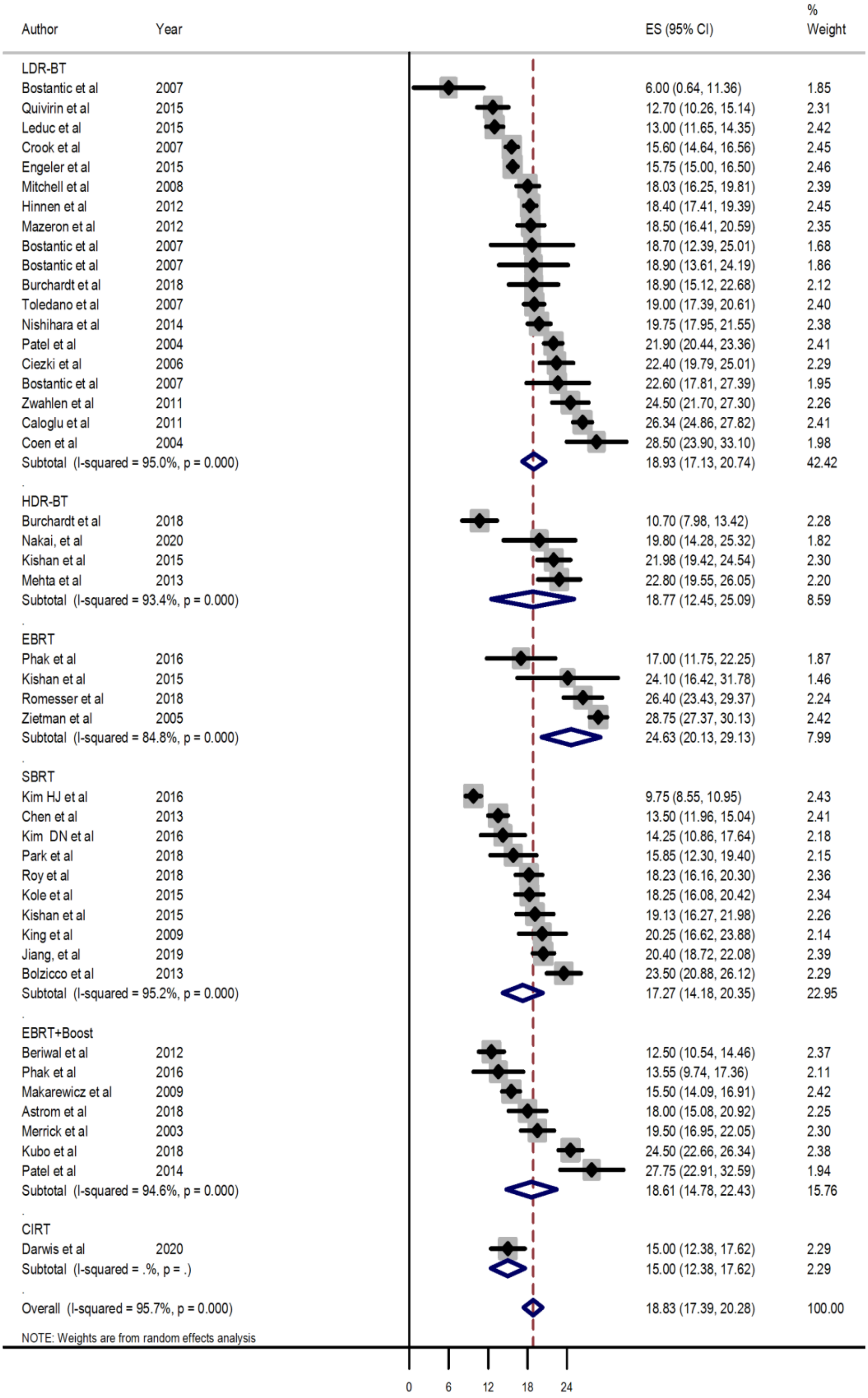

Figure S2. Meta-analysis of the time to occurrence of prostate-specific antigen (PSA) bounce after radiotherapy. ES, effect size; CI, confidence interval; LDR-BT, low dose-rate brachytherapy; HDR-BT, high dose-rate brachytherapy; EBRT, external beam radiotherapy; SBRT, stereotactic body radiotherapy; CIRT, carbon ion radiotherapy.

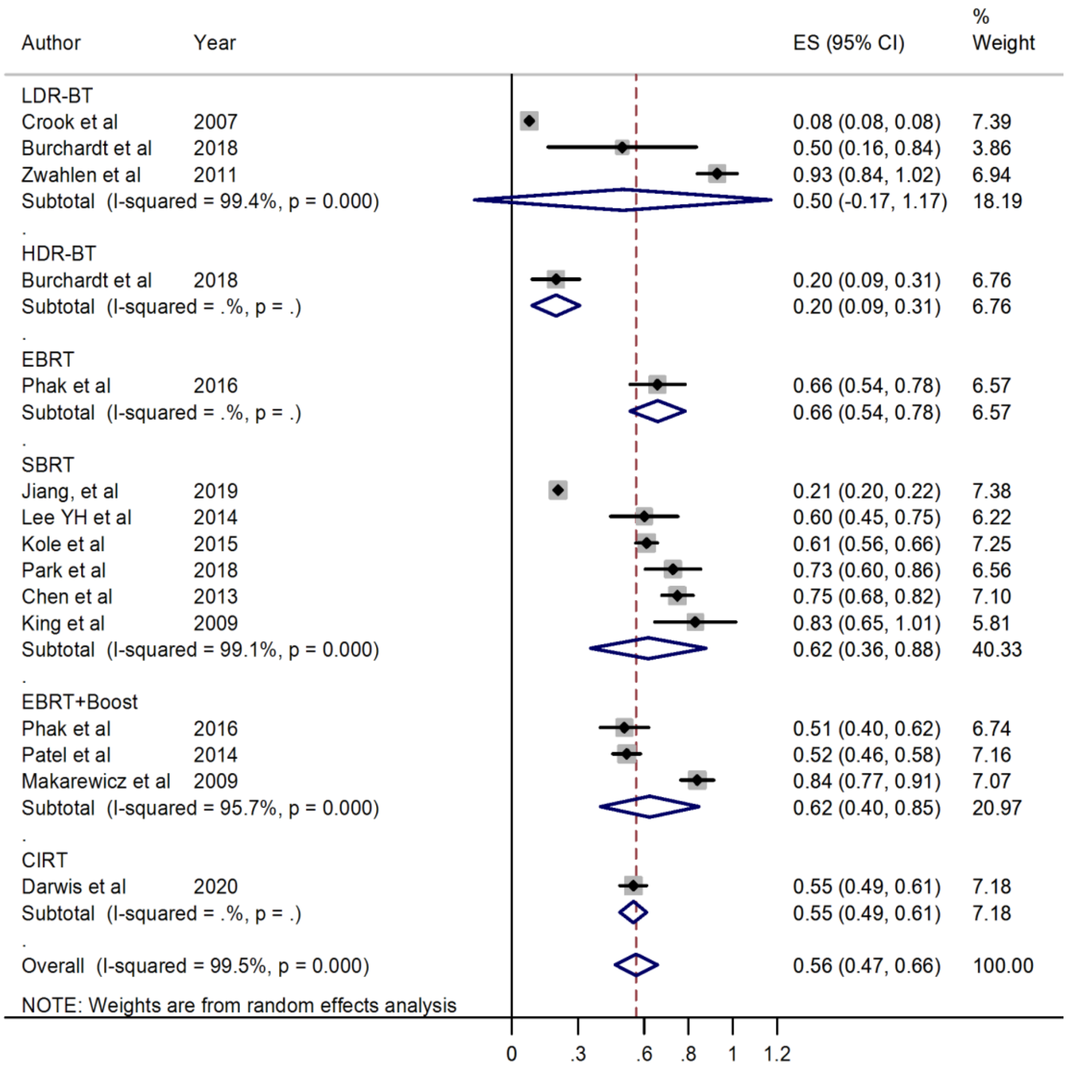

Figure S3. Meta-analysis of prostate-specific antigen (PSA) nadir values after radiotherapy. ES, effect size; CI, confidence interval; LDR-BT, low dose-rate brachytherapy; HDR-BT, high dose-rate brachytherapy; EBRT, external beam radiotherapy; SBRT, stereotactic body radiotherapy; CIRT, carbon ion radiotherapy.

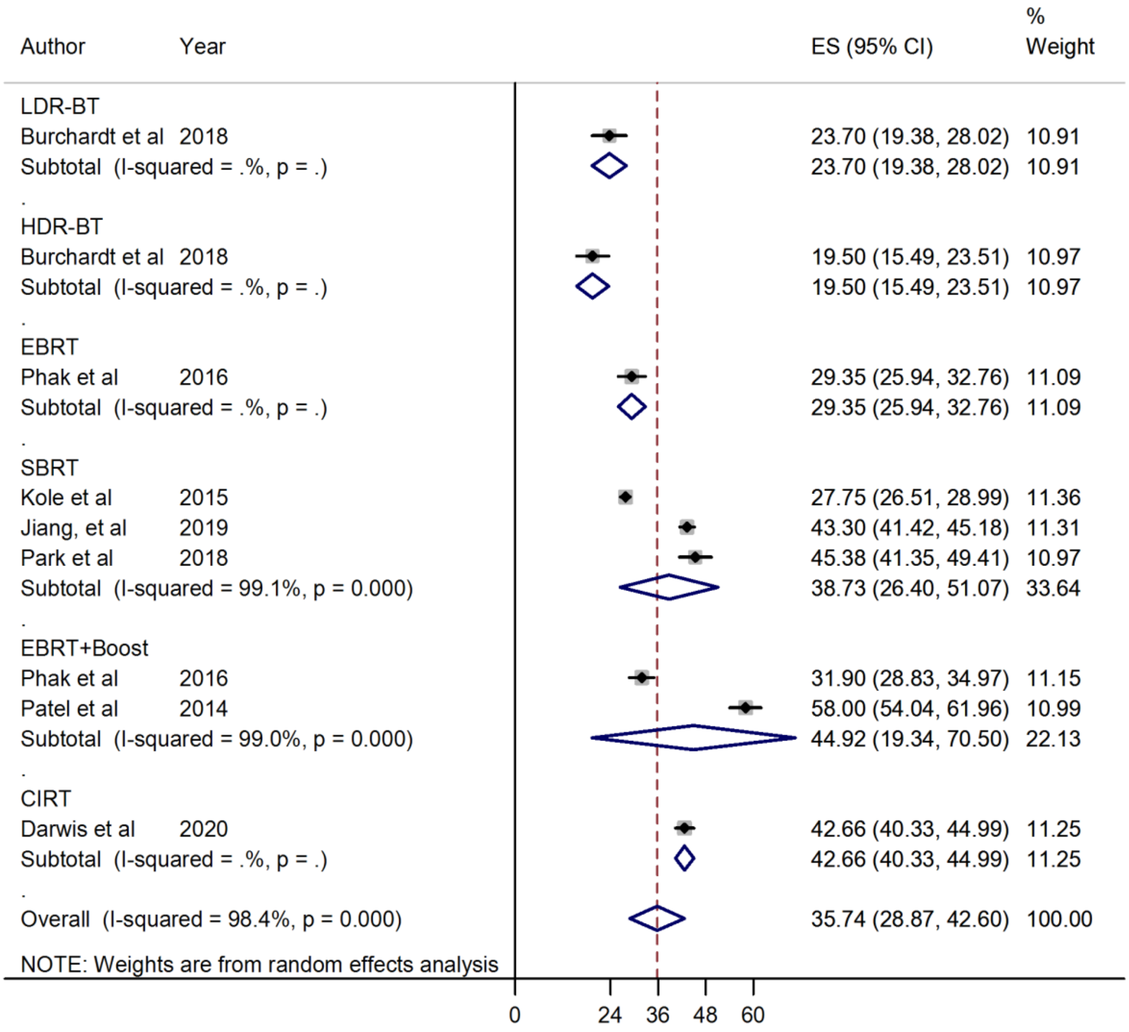

Figure S4. Meta-analysis of the time to prostate-specific antigen (PSA) nadir after radiotherapy. ES, effect size; CI, confidence interval; LDR-BT, low dose-rate brachytherapy; HDR-BT, high dose-rate brachytherapy; EBRT, external beam radiotherapy; SBRT, stereotactic body radiotherapy; CIRT, carbon ion radiotherapy.
